# Genetic Variants in RANK and OPG Could Influence Disease Severity and Bone Remodeling in Patients with Early Arthritis

**DOI:** 10.3390/life14091109

**Published:** 2024-09-03

**Authors:** Ana Triguero-Martínez, Marisa Pardines, Nuria Montes, Ana María Ortiz, Alba de la Iglesia-Cedeira, Cristina Valero-Martínez, Javier Martín, Isidoro González-Álvaro, Santos Castañeda, Amalia Lamana

**Affiliations:** 1 Rheumatology Department, Hospital Universitario La Princesa, Instituto de Investigación Sanitaria La Princesa (IIS-IP), 28006 Madrid, Spain; ana6n92@gmail.com (A.T.-M.); marisavilladebiar@hotmail.com (M.P.); nuria.montes.casado@gmail.com (N.M.); lanult@yahoo.es (A.M.O.); cristina.valmart@gmail.com (C.V.-M.); isidoro.ga@ser.es (I.G.-Á.); 2 Cell Biology Department, Facultad de Biología, Universidad Complutense de Madrid, 28040 Madrid, Spain; albdelai@ucm.es; 3 Institute of Parasitology and Biomedicine “Lopez-Neyra”, CSIC, 18016 Granada, Spain; javiermartin@ipb.csic.es

**Keywords:** early arthritis, single-nucleotide polymorphisms, bone remodeling, bone mineral density, severity, osteoimmunology

## Abstract

The aim of this study was to identify single-nucleotide polymorphisms (SNPs) in bone remodeling-related genes associated with disease severity and bone mineral density (BMD) in early arthritis (EA) patients. For this purpose, the genotyping of 552 SNPs located in gene regions of semaphorins 4b, 4d, 4f, DKK1, 2 and 3, sclerostin, OPG, RANK and RANKL was performed using Immunochip from Illumina Inc. in 268 patients from the Princesa Early Arthritis Register Longitudinal (PEARL) study. Measurements of BMD and disease activity were chosen as outcome variables to select SNPs of interest. The relationships of SNPs with the BMD of the forearm, lumbar spine and hip (Hologic-4500 QDR) were analyzed by linear regression adjusted for age, sex, body mass index and presence of anti-citrullinated peptide antibodies (ACPAs). The association of each SNP with activity variables was analyzed by linear regression, logistic regression or ordered logistic regression according to the variable, and multivariate models were adjusted for potentially confounding variables, such as age, sex and presence of ACPAs. These analyses showed that four SNPs located in the genes coding for RANK (*TNFRSF11A*) and OPG (*TNFRSF11B*) were significantly associated with clinical variables of severity. SNP rs1805034 located in exon 6 of *TNFRSF11A*, which causes a non-synonymous (A/V) mutation, showed significant association with BMD and therefore may be considered as a possible biomarker of severity in RA patients. SNPs in the OPG gene showed an association with serum OPG levels and predicted disease activity after two years of follow-up.

## 1. Introduction

Rheumatoid arthritis (RA) is the most common chronic autoimmune rheumatic disease causing polyarthritis, with a prevalence of approximately 1% in the general population [1]. RA is characterized by chronic infiltration of immune cells to the synovial membrane. These infiltrated cells cause chronic production of proinflammatory cytokines (TNF-α, IL-1β and IL-6) and metalloproteinases that usually leads to irreversible joint destruction due to cartilage degradation and bone erosion [2]. The etiology of this disease still remains to be elucidated, although it is known that the interaction of genetic and environmental (advanced age, smoking and overweightness, among others) risk factors can be involved in RA development [3].

At the beginning of this century, Arron et al. introduced the term “osteoimmunology” to describe the relationship between the skeletal and immune systems, highlighting the connection between abnormal bone metabolism and immune system disorders [4]. As a result, this concept has become essential for understanding the pathogenesis of skeletal and joint diseases related to abnormal immune responses. Many biochemical mediators once thought to be exclusively immune-related, such as proinflammatory cytokines and transcription factors, are now known to be shared by both systems [5,6,7,8,9,10,11].

RA is a disease where inflammation of the synovium leads to bone destruction in multiple joints. Osteoclasts and mononuclear cells are key cell populations responsible for bone loss during RA [12]. In normal conditions, there is a balance in bone tissue between bone formation by osteoblasts and bone resorption by osteoclasts, supporting the final bone homeostasis of the subject. However, this balance is disrupted in pathological conditions when bone resorption exceeds bone formation or vice versa [13]. Accordingly, investigation of the molecular mechanisms underlying bone destruction in RA has been one of the main driving forces behind the evolution of osteoimmunology [14].

Multiple studies have shown that the ligand/cognate receptor pair formed by receptor activator of nuclear factor kappa-B ligand (RANKL) and receptor activator of nuclear factor kappa-B (RANK), as well as the decoy receptor osteoprotegerin (OPG), play a crucial role in controlling the relationship between bone homeostasis, the immune system, inflammation and other systems under pathophysiological conditions [15]. OPG competitively binds to RANKL and blocks its binding to RANK, thereby preventing osteoclast maturation and differentiation and consequently blocking osteoclastic bone resorption [15].

The dysregulation of the RANKL-RANK-OPG axis at the bone level has been described in the context of RA pathology because this pathway provides critical signals that control intercellular communication between bone-forming osteoblasts and bone-resorbing osteoclasts [16].

Genome-wide association studies (GWASs) in RA patients have identified approximately 101 loci associated with increased susceptibility to RA [17]. However, the individual contribution of each locus is minimal, and there is limited information on the significance of interactions between these genetic factors [3]. Remarkably, even less is known about the role of genetic variants in RA severity. Consequently, numerous studies have concentrated on identifying genetic variants, especially single-nucleotide polymorphisms (SNPs), in molecules that play a crucial role in the pathophysiology of RA or in factors related to disease severity.

Due to the importance of the molecules associated with bone homeostasis, especially the RANKL-RANK-OPG axis, in the progression and severity of RA, several studies have focused on studying the genetic variability of the genes coding for these molecules in different populations [18]. Therefore, in our study, we have chosen the molecules of the RANK/RANKL/OPG axis and the Wnt/DKK1/sclerostin pathway, because these molecules play a key role in controlling the relationship between bone homeostasis, the immune system and inflammation on the RA disease spectrum and other osteoimmune diseases [19,20]. In fact, there are several studies that have related these molecules and some correlated genes to the course and severity of RA [18,21,22].

Taking all these data into consideration, the aim of this study was to evaluate the association of RA with different genetic variants of key molecules related to bone metabolism such as OPG or RANK and to assess whether they are also related to disease severity in patients with recent-onset arthritis.

## 2. Materials and Methods

### 2.1. Study Population

The present study was performed with data and samples from the Princesa Early Arthritis Register Longitudinal (PEARL) study that included patients referred to the Early Arthritis (EA) Clinic of a tertiary university hospital in Spain. The registry protocol included the collection of sociodemographic, clinical, therapeutic and laboratory data, as well as biological samples collected at 4 visits during a two-year follow-up period (0, 6, 12 and 24 months). At the 24-month visit, all patients were classified as RA if they met the 1987 ACR classification criteria and/or 2010 ACR/EULAR classification criteria [23,24] or as undifferentiated arthritis (UA) as described by Verpoort et al. [25]. Basically, all patients included were over 18 years old and had arthritis of two or more joints for at least 4 weeks and less than 12 months’ duration. Patients with other defined diagnoses (connective tissue diseases, psoriatic arthritis, gout or osteoarthritis) were excluded from the study. Likewise, patients with moderate/severe chronic kidney damage were excluded from the study due to the influence that renal failure could have on the determination of OPG and RANKL serum levels [26]. Patients with cancer were also excluded from the study. A more detailed description of the PEARL protocol has been previously published [27].

Patients included in the PEARL study have protocolized Dual X-ray densitometry (DXA) of the hand, hip and lumbar spine (LS) at the baseline, 6-, 12- and 24-month visits, although the data analyzed in this study are only from the baseline visit (Table 1).

The patients included in this study are 97% Spanish, and there are 6 patients from South America (Latin/Hispanic) and 2 from Eastern Europe (Slavic), who were not excluded because the allele frequencies of the SNPs analyzed were similar to those of the Spanish population.

The present study was conducted according to the principles expressed in the Helsinki Declaration of 1983, updated at the Seoul, Korea, meeting in 2008, and it was approved by the Research Ethics Committee of Hospital Universitario de La Princesa (PI-518; 28 March 2011). All patients included signed a written informed consent before their participation in the study.

### 2.2. Determination of Anti-Citrullinated Cyclic Peptide Antibodies (ACPAs)

ACPA determination was performed by enzyme immunoassay with second-generation anti-citrullinated cyclic peptide enzyme immunoassay (EIA; Euro-Diagnostica AB; now Svar Life Science, Malmö, Sweden) (positive >50 IU/mL) until October 2010 and thereafter with a third-generation EIA (QUIA; Euro-Diagnostica AB; Svar Life Science, Malmö, Sweden) (positive >40 IU/mL).

### 2.3. Serum RANKL, OPG and IL-6 Measurements

Serum samples were obtained at each visit of the PEARL study. Samples were immediately centrifuged and the cell-free supernatant frozen at –80 °C.

Serum OPG and RANKL levels were assessed using an enzyme-linked immunosorbent assay from R&D Systems (Minneapolis, MN, USA) and Immundiagnostik/Apotech (Epalinges, Switzerland), respectively, following the manufacturer’s instructions. IL-6 serum levels were routinely measured using the Human IL-6 Quantikine high-sensitivity enzyme-immune assay from R&D Systems Europe Ltd. (Abingdon, UK) in samples from those patients with at least three visits along the two-year follow-up (769 visits; 3.8 visits per patient).

The optical density of each well in duplicate was determined using a microplate reader set at 450 nm with a wavelength correction at 570 nm.

### 2.4. Genotyping

The SNPs located in genes selected because of their involvement in bone remodeling were genotyped in 268 patients from the PEARL study using an Illumina iScan System Immunochip array (Illumina Inc., San Diego, CA, USA). This chip array is a high-throughput, high-density array designed to fine-map immune-related loci, which include 196,524 SNPs with genetic positions according to the NCBI build 36 (hg18) map (Illumina manifest file Immuno_BeadChip_11419691_B.bpm). Immunochip’s raw data were filtered using PLINK v1.07 software. Samples were discarded when they showed <90% of successfully called SNPs, and SNPs were discarded according to the following criteria: when call rates were <95% (low quality), minor allele frequencies (MAF) <0.1, *p*-value for Hardy–Weinberg equilibrium deviation <0.001. The inclusion of 6 patients from South America (Latin/Hispanic) and 2 from Eastern Europe (Slavic) was due to the fact that the allelic frequencies of the SNPs analyzed were similar in these populations.

### 2.5. Variables

To analyze disease activity, we used the DAS28 and HUPI scores [28,29,30]. Disease activity at the end of the two-year follow-up was categorized using the DAS28 cut-off values proposed by Prevoo et al. (<2.6, remission; 2.6 to 3.2, low disease activity; >3.2 to 5.1, moderate disease activity; >5.1, high disease activity) [28]. Similarly, the HUPI cut-off points to determine RA activity are ≤2, remission; >2 and ≤5, mild activity; >5 and ≤9, moderate; and >9 high activity [2,30]. We described the clinical parameters between the groups according to activity levels categorized according to DAS28 in Table 2.

In addition, two dichotomous variables were generated according to DAS28-based categorization: (i) “Remission”, in which patients were classified as “Remission” if they had DAS28 <2.6 at the 24-month visit or as “no remission” if they had DAS28 ≥2.6; (ii) “Therapeutic target” when patients met the target (if DAS28 at 24 months was ≤3.2) or “No target” when patients did not meet the therapeutic target (DAS28 at 24 months >3.2).

Diagnosis at the end of follow-up in the PEARL population was expressed as a dichotomous variable (UA or RA), as described in the Section 2.1.

For the radiographic variables studied, plain radiographs of the non-dominant hand were taken in EA patients selected to perform a computerized study of the diaphyseal bone mass of the second to fourth metacarpals (MCs) of the non-dominant hand by means of the Pronosco© system based on the digital X-ray of both hands of all the patients included in the study.

Subsequently, a Dual X-ray densitometry (DXA) was performed in order to evaluate BMD. The measurements were performed with a HOLOGIC© 4500W Elite series densitometer (Hologic©, Bedford, MA, USA). The densitometer was calibrated daily using an anthropomorphic LS phantom [coefficient of variability (CV) obtained in the spine in the last year: 0.46%]. In addition, a conventional DXA of the non-dominant hand was performed with the subregion program to determine the bone mass in the second to fifth metacarpophalangeal (MCP) joints, as well as at the level of the MC diaphysis of that hand as previously described [31,32]. Absolute BMD results are expressed as g/cm^2^.

### 2.6. Statistical Analysis

Statistical analyses were performed using STATA 14 for Windows (Stata Corp LP, College Station, TX, USA). Continuous variables were described using mean and standard deviation (SD). Most quantitative variables followed a non-normal distribution, so they were represented as median and interquartile range (IQR), and the Mann–Whitney or Kruskal–Wallis test was used to analyze significant differences. Dichotomous and categorical variables were described using proportions, and the χ^2^ or Fisher’s exact test was used to compare categorical variables. Cuzick’s test, an extension of the Wilcoxon rank-sum test, was used to determine the statistical significance of the distribution trend across ordered groups in variables such as visits or level of disease activity. Statistical significance was considered at *p* < 0.05.

#### Multivariate Analyses

To filter those SNPs that could influence disease activity in EA patients, we first fitted a multivariate analysis for DAS28 using generalized linear models analyzed by patient and visits using the “xtgee“ command of STATA. Population-averaged generalized estimating equations were first modeled by adding all variables with a *p*-value < 0.15 to the bivariate analysis. The final models were constructed by quasi-likelihood estimation based on the independence model information criterion and Wald tests, removing all variables with *p*-value > 0.15. Once the best model was obtained in the previous phase, the genotype variable for each of the SNPs located in the genes of interest was forced into the model described previously.

In addition, we constructed six additional multivariate models: two ordered logistic regression models using the “ologit” command of STATA for the activity variables categorized into four levels (using DAS28 and HUPI activity indexes) and two logistic regression models using the “logit command” of STATA for the dichotomous variables “Remission” and “Therapeutic target” defined above. The criteria for generating the final models followed the same backward-stepwise selection, eliminating variables with *p* > 0.15 and forcing the genotype of each SNP in the final model, as described above.

The effect of the SNPs on BMD was evaluated by linear regression models (“regress” command of STATA) using as dependent variables BMD in LS, in second to fifth MC diaphysis of the non-dominant hand, total non-dominant hand, femoral neck, total femur and mid, distal and ultradistal radius, all measured at the baseline visit.

## 3. Results

### 3.1. Screening of SNPs in Bone Metabolism Genes Related to Relevant Clinical Parameters in RA

In this study, we examined data from 268 early arthritis (EA) patients for whom we have genotyped 196,524 polymorphisms using the Illumina iScan System Immunochip array (Illumina Inc., San Diego, CA, USA). This array targets regions of genes involved in immune system regulation [29]. Initially, we searched for SNPs within genes related to bone metabolism that were represented in the iCHIP. We selected 553 SNPs in the following genes: TNF receptor superfamily member 11A or RANK (*TNFRSF11A*), TNF superfamily member 11 or RANKL (*TNFSF11*), TNF receptor superfamily member 11B or osteoprotegerin (OPG) (*TNFRSF11B*), sclerostin (*SOST)*, semaphorins (*SEMA3A*, *SEMA4B*, *SEMA4D*, *SEMA4F*, and *SEMA6D*), calcitonin receptor (*CALCR*) and DKK1.

After excluding SNPs with an MAF < 0.1 and those in complete linkage disequilibrium (LD) with another SNP, we analyzed 169 SNPs. Of these, 162 were located in intergenic regions near the selected genes, and only 7 were in coding regions, introns or 3′ untranslated regions (3′UTR).

Given the relationship between bone mass at disease onset and disease severity previously described in RA [33], we analyzed the association between the genotypes of the 169 SNPs and outcome variables related to disease activity (DAS28 or HUPI) and BMD measured at the hip, femoral head, LS, distal radius and hands.

**Figure 1:**
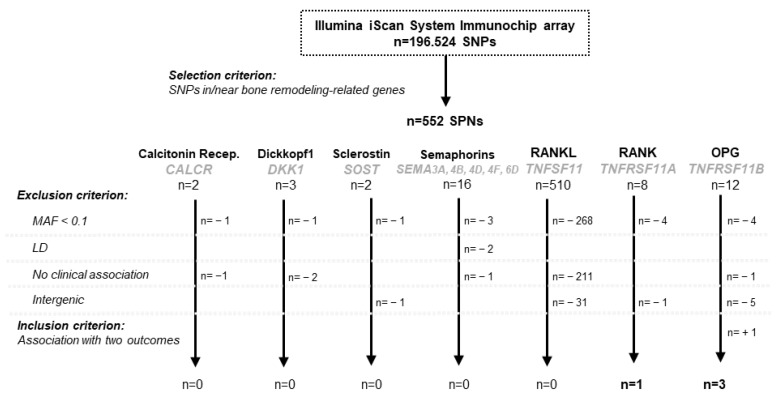
The flow chart shows the SNP selection process from the 552 SNPs included in the iCHIP with the genes of interest. Inclusion and exclusion criteria for SNP selection are shown in the figure. The name of each protein is shown in bold and below it, the name of the gene (gray and italics). LD, linkage disequilibrium; MAF, minor allele frequency; SNPs, single-nucleotide polymorphisms.

We identified 40 SNPs associated with several clinical parameters (Supplementary Table S1). Among them, only three SNPs were located within gene regions: *TNFRSF11B* (rs3134058 and rs10505346) and *TNFRSF11A* (rs1805034). Additionally, the intergenic SNP rs4355801, located near *TNFRSF11B*, was found to be associated with both BMD and disease activity (Figure 1).

### 3.2. SNPs in the OPG Gene Are Associated with Serum OPG Levels and Predict Disease Activity after Two Years of Follow-Up

**Figure 2:**
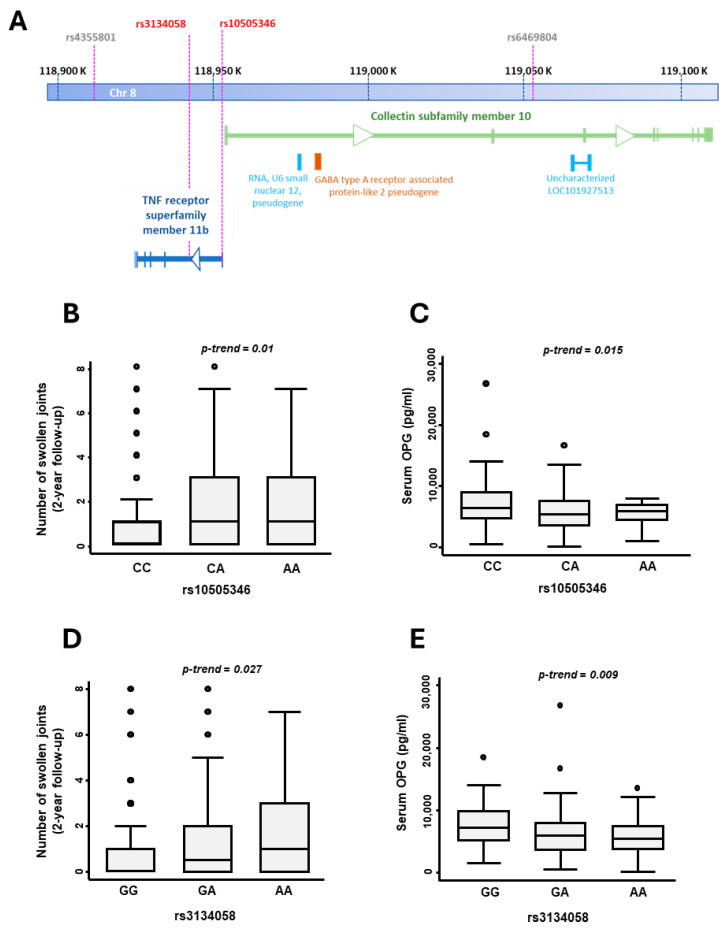
*TNFSF11B* genetic variants are associated with OPG serum levels and the number of swollen joints at the end of the two-year follow-up. (**A**) The scheme shows the *TNFSF11B* gene located on chromosome 8 and the two SNPs located in intronic regions, rs3134058 and rs10505346. (**B**) The number of swollen joints at the end of the two-year follow-up according to the genotypic distribution of rs10505346 (genotypes indicated in the figure). (**C**) The panel shows OPG serum levels (pg/mL) according to rs1050505346 genotypes. (**D**) The panel shows the number of swollen joints at the end of the two-year follow-up according to the genotypic distribution of rs3134058 (genotypes indicated in the figure). (**E**) The panel shows serum OPG levels (pg/mL) according to rs3134058 genotypes. In panels B to E, data are shown as interquartile range (p75 upper edge of box, p25 lower edge, p50 midline) as well as p95 (line above box) and p5 (line below box). Dots represent outliers. The statistical significance of the associations was analyzed by the Cuzick’s test (STATA 14).

The SNPs rs10505346 and rs3134058, located in the first intron of the *TNFRSF11B* gene (Figure 2A), showed a significant association with the number of swollen joints at the end of the two-year follow-up (*p* = 0.01 and *p* = 0.027, respectively; Figure 2B,D). To analyze their effect on disease activity, we performed a multivariate logistic regression, categorizing disease activity at two years according to the HUPI activity index [29]. The best model, which included the variables age, final diagnosis and positivity for ACPAs, showed that the presence of the AA genotype for rs10505346 was associated with increased activity at two years (Supplementary Table S2). Additionally, a multivariate analysis of DAS28 [nested by patient and visit, accounting for the effect of medication and final diagnosis (RA or UA)] over the total follow-up period (four visits) revealed that patients carrying the AA genotype for rs10505346 had higher disease activity throughout the follow-up (β-Coeff = 0.50, *p* = 0.04; Table 3).

Patients who carried the AA genotype for rs3134058 also showed a significant association with higher disease activity at the end of the two-year follow-up, as indicated by the logistic regression of DAS28 categorized as remission-mild vs. moderate-high, defined as “therapeutic target vs. no target” in Section 2 (β-Coeff = 0.86, *p* = 0.038; Table 4).

In high bone turnover disorders, the up-regulation of *OPG* could be a compensatory mechanism to limit bone erosion [34]. Since patients carrying minor alleles at rs3134058 and rs10505346 in the OPG gene had a worse prognosis, we investigated whether these SNPs might be altering OPG expression levels. The results showed that those patients who carried the minor alleles of genetic variants rs10505346 and rs3134058 exhibited reduced serum OPG levels throughout the follow-up (*p* = 0.015 and *p* = 0.009, respectively; Figure 2C,E).

### 3.3. The SNP rs4355801, Located near the TNFRSF11B Gene, Is Associated with Good Prognosis and Higher OPG Levels during Follow-Up

**Figure 3:**
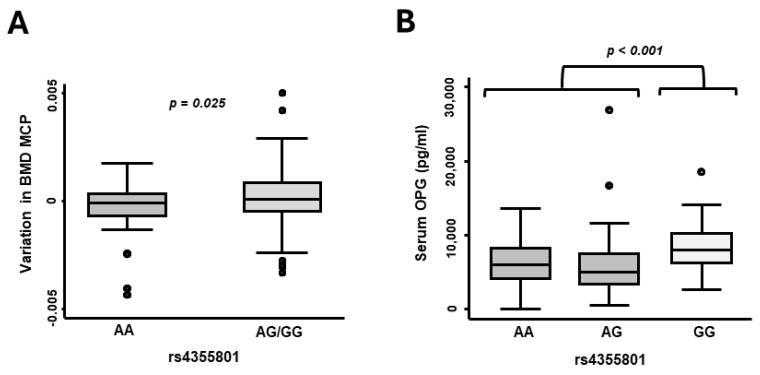
The SNP rs4355801 located near *TNFSF11B* is associated with variation in bone mineral density (BMD) as well as with serum OPG levels. (**A**) The graph shows variation in BMD (BMD measurement at the 24-month visit minus basal BMD measurement) according to the genotypic distribution of rs4355801 variants (genotypes indicated in the figure) (*p* = 0.025 by the Mann–Whitney test). (**B**) The graph shows the serum levels of OPG (pg/mL) according to the genotypes of rs4355801 (*p* < 0.001 by the Kruskal–Wallis test). BMD, bone mineral density; MCP, metacarpophalangeal joints. Data are shown as interquartile range (p75 upper edge of box, p25 lower edge, p50 midline) as well as p95 (line above box) and p5 (line below box). Dots represent outliers.

A multivariate analysis of DAS28 throughout the follow-up period accounting for the effect of medication and final diagnosis (RA or UA) revealed that patients with the GG genotype of rs4355801 exhibited lower disease activity (β-Coeff= −0.961, *p* = 0.014; Table 5). Additionally, patients who carried the GG genotype showed a greater improvement in BMD from baseline to final measurement (∆-BMD) compared to AA patients, indicating a stronger bias towards bone regeneration (*p* = 0.025; Figure 3A). Moreover, these patients had significantly higher levels of serum OPG than those with other genotypes (*p* < 0.001; Figure 3B).

### 3.4. Combined Genotypic Model of SNPs Related to TNFRSF11B Gene Shows Significant Association with Bone Mass Gain and Serum OPG Levels during Follow-Up

**Figure 4:**
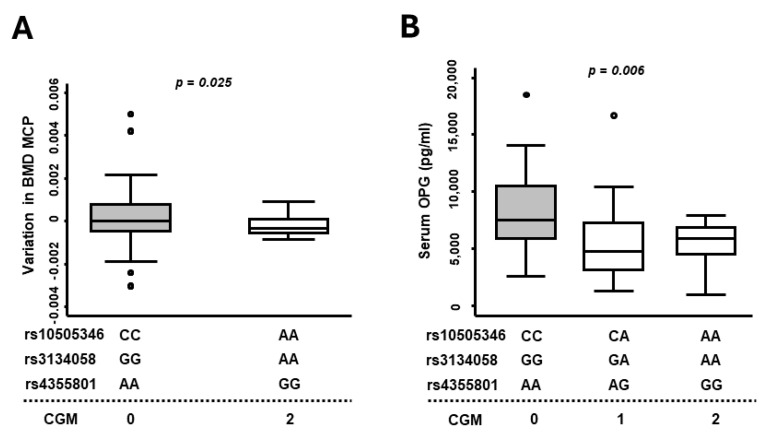
The Combined Genotypic Model (CGM) for SNPs located near or in the *TNFSF11B* gene is associated with the variation in bone mineral density (BMD) as well as with serum OPG levels. (**A**) The graph shows the variation in BMD at MCP joints (BMD measurement at the 24-month visit minus basal BMD measurement) according to the CGM performed with rs10505346, rs3134058 and rs4355801 genotypes (combination indicated in the figure) (*p* = 0.025 by the Mann–Whitney test). (**B**) The graph shows the serum levels of OPG (pg/mL) according to the CGM (*p* = 0.006 by the Kruskal–Wallis test). BMD, bone mineral density; MCP, metacarpophalangeal joints. Data are shown as interquartile range (p75 upper edge of box, p25 lower edge, p50 midline) as well as p95 (line above box) and p5 (line below box). Dots represent outliers.

Based on the above results, we decided to generate a Combined Genotypic Model (*CGM*) of the SNPs rs10505346, rs3134058 and rs4355801 to improve the statistical power to predict prognosis. In this way, we performed a comparison among the combination of the alleles that had been associated with higher serum levels of OPG versus those that had been associated with the lowest levels (*CGM* = 0 for the combination CC-rs10505346, GG-rs3134058 and GG-rs4355801; *CGM* = 2 for the combination AA-rs10505346, AA-rs3134058 and AA-rs4355801 and *CGM* = 1 for heterozygotes of each SNP). Using this tool, it was confirmed that the combination of genotypes was associated with OPG serum levels (n = 117, *p* = 0.006, Figure 4B), and it was also observed that patients carrying alleles associated with higher OPG serum levels (CGM = 0) showed higher increases in BMD at MCP joints (∆-BMD) during follow-up compared to patients associated with lower serum OPG levels (*p* = 0.025; Figure 4A). Using the *CGM*, we performed a multivariate analysis of the DAS28 throughout the follow-up period and we observed that patients with *CGM* = 2 had a tendency toward greater activity compared to *CGM = 0*. Additionally, the HUPI index at two years of follow-up was also significantly higher in patients with *CGM* = 2 compared to *CGM* = 0 (beta coeff = 1.9, *p* = 0.01, Supplementary Table S3).

### 3.5. The CC-rs1805034 Genotype of the RANK Gene Is Associated with Low BMD Values and High IL-6 Levels at the Baseline Visit

**Figure 5:**
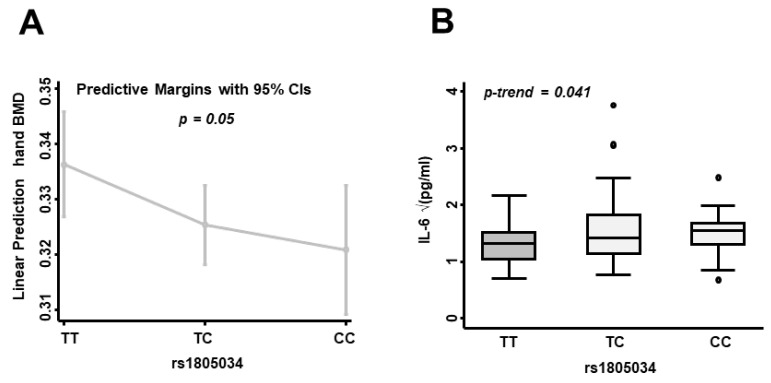
The GG-rs1805034 genotype of the RANK gene is associated with low bone mineral density (BMD) values and high IL-6 levels at the baseline visit. (**A**) The graph shows a linear prediction of hand BMD after multivariable model fitting adjusted for age, sex and body mass index using the “margins” command of STATA. (**B**) The graph shows IL-6 levels (pg/mL) according to the distribution of rs1805034 genotype variants after normalizing the variable by the square root. Data are shown as interquartile range (p75 upper edge of box, p25 lower edge, p50 midline) as well as p95 (line above box) and p5 (line below box). Dots represent outliers. The statistical significance of the distributions was calculated by the Cuzick’s test (STATA 14).

One of the SNPs identified in the initial screening as being associated with BMD parameters was rs1805034, located in the sixth intron of the *TNFRSF11A* gene. Analysis adjusted for age, sex and body mass index showed that patients who carried the minor alleles of this SNP had lower BMD at the baseline visit, measured in the hand and distal radius (β-Coeff = −0.015, *p* = 0.046, and β-Coeff = −0.02, *p* = 0.047, respectively). Furthermore, a multivariate analysis nested by patient and visit showed that the presence of minor alleles at rs1805034 was significantly associated with BMD measured in the hand (TC-rs1805034: β-Coeff = −0.012, *p* = 0.032 and CC-rs1805034: β-Coeff = −0.017, *p* = 0.018; Table 6) and MCP joints (TC-rs1805034: β-Coeff = −0.011, *p* = 0.059 and CC-rs1805034: β-Coeff = −0.015, *p* = 0.039) (Figure 5A).

Patients who carried the minor alleles at rs1805034 also had higher IL-6 levels at the baseline visit (Figure 5B, *p* = 0.041). However, this SNP did not show any association with serum RANKL levels, nor with OPG levels or with the RANK/OPG ratio.

## 4. Discussion

Osteoimmunology is a discipline that studies the close relationship between the bone and the immune system [4,6,7,8,9], the RANKL/RANK/OPG axis being one of the main lines of communication and interconnection between both systems [19,20]. Our study shows that the alleles associated with low levels of OPG in the *TNFRSF11B* gene predict more disease activity at two years of follow-up in a register of patients with EA. Furthermore, the SNP rs4355801, located near the *TNFRSF11B* gene, is associated with good prognosis and higher OPG levels during follow-up in the same cohort of patients. Finally, the CC genotype of rs1805034, in the RANK gene, is associated with low BMD values and high IL-6 levels at the baseline visit of patients included in this study.

We identified 40 SNPs associated with several clinical parameters of interest. Among them, only three were located within gene regions: *TNFRSF11B* (rs3134058 and rs10505346) and *TNFRSF11A* (rs1805034). Interestingly, the intergenic SNP rs4355801, located near *TNFRSF11B*, was found to be associated with both BMD and disease activity outcomes.

OPG (*TNFRSF11B*) plays a crucial role in bone biology through the regulation of osteoclastogenesis and bone homeostasis, and it is the target of several therapeutic agents. Nevertheless, current data on the relationship between the RANKL/RANK/OPG genes and bone mass or disease activity in patients with RA are very limited [21,35,36,37].

A GWAS analyzing the most promising of 314,075 SNPs in 2,094 women in the UK found an association between BMD and two SNPs: rs4355801 on chromosome 8, near the *TNFRSF11B* gene, also found in our study, and rs3736228 on chromosome 11, in the LRP5 (lipoprotein-receptor-related protein) gene [35]. In addition, three SNPs near the *TNFRSF11B* gene were associated with decreased BMD and increased risk of osteoporosis. In particular, rs4355801, which is located in the 3’ UTR of the gene, remained associated with BMD variations with genome-wide significance [35]. The GG genotype of rs4355801 has been reported to be associated with higher BMD and increased expression of OPG in patients with osteoporosis. Conversely, the AA genotype of this SNP has been linked to lower BMD in LS and femoral neck, as well as to an increased risk of developing osteoporosis [35].

Indeed, the variant with the greatest association (the risk allele A in rs4355801) was the most common in that population (79%). In fact, each copy of the A risk allele was associated with a decrease in LS-BMD by 0.09 SD in the replication cohorts [35]. In our initial analyses, rs4355801 showed an effect on both disease activity and BMD, prompting us to select it for further investigation despite its intergenic position. In accordance with published data, in our register of patients with EA, the risk allele A was associated with less bone mass gain at MCP joints at the end of follow-up. Likewise, carriers of the A allele had lower serum OPG levels during follow-up. Conversely, in our population of patients with EA, the minor allele G was associated with improvement in BMD and also with higher levels of OPG during follow-up. Similarly, in the results published by Richards et al., the allele G in rs4355801 was associated with higher BMD and higher gene expression of *TNFRSF11B* [35].

Interestingly, two other SNPs, located in the first intron of *TNFRSF11B* (rs10505346 and rs3134058), showed a significant association with the number of swollen joints at the end of follow-up in our study. With the aim of studying the association between SNPs and disease activity, we categorized the activity of our patients according to the HUPI score and found that the AA genotype of rs10505346 was associated with increased activity at two years. Additionally, a multivariate analysis of DAS28 over the total follow-up revealed that patients who carried the AA genotype of rs10505346 had higher disease activity throughout the follow-up. Likewise, patients carrying the AA genotype of rs3134058 showed a significant association with higher disease activity at the end of the two-year follow-up. Since patients who carried minor alleles at rs3134058 and rs10505346 in the OPG gene had a worse prognosis, we investigated whether these SNPs might be altering OPG gene expression levels. The results showed that those patients carrying the minor alleles of the genetic variants rs10505346 and rs3134058 exhibited reduced serum OPG levels throughout the follow-up. These data reinforce our previous findings regarding the rs4355801 SNP near the *TNFRSF11B* gene and confirm OPG as a key factor in the intricate relationship between bone mass, OPG levels and disease activity in EA patients. Unlike our results, a meta-analysis conducted by Wang et al. revealed a significantly higher circulating OPG level in RA patients, and it was influenced by race, disease duration, body mass index and DAS28 [38]. Nevertheless, one of the main limitations of this work is that part of the included studies did not report DAS28 values; thus, the association between circulating OPG level and DAS28 could not be effectively assessed [38].

To evaluate the combined effect of the SNPs located near or in the OPG gene on the prognostic variables, we generated a combined genotype model (CGM). The model significantly corroborated that patients carrying A alleles for rs10505346, A alleles for rs3134058 and G alleles for rs4355801 showed lower serum OPG levels. Furthermore, the double dose of these alleles was significantly associated with bone mass loss during disease follow-up and higher disease activity at two years of follow-up. All of this suggests that the generation of predictive genetic indices based on the combination of alleles can improve the statistical power of the analyses. The CGM has allowed us to establish the association between serum levels of OPG, bone mineral density and disease activity, even having a lower number of patients due to the stratification of the analysis. These findings indicate that the generation of predictive genetic indices based on the combination of alleles can improve the prediction of disease severity.

Finally, we studied the role of RANK in bone remodeling, BMD and disease activity in our cohort of patients with EA. RANK is a membrane receptor expressed in osteoclasts and osteoclast precursors that regulates the differentiation, activation and survival of these cell types [10,21,39,40]. These functions could link RANK to a worse RA prognosis. In the present work, one of the SNPs identified as being associated with BMD parameters was rs1805034, located in the sixth exon of the *TNFRSF11A* gene. In fact, patients who carried the minor alleles of this SNP had lower BMD at the baseline visit, measured in the hand and distal radius. Furthermore, a multivariate analysis nested by patient and visit showed that the presence of the genotype CC at rs1805034 was significantly associated with BMD decrease in the hand and MCP joints. In addition, patients who carried the minor allele (“C”) of rs1805034 had higher IL-6 levels at the baseline visit. These increased levels of IL-6 could be associated with a worse prognosis and outcomes, since IL-6 is a powerful proinflammatory cytokine involved in increased inflammatory burden, increased bone remodeling and secondary bone destruction. Nevertheless, rs1805034 C allele is a missense variation that causes a change in the amino acid sequence of RANK from a glutamic acid to an alanine. Therefore, further studies would be needed to study the functional effect of this mutation.

These results are in agreement with those observed by Mohamed et al. in a cohort of 172 postmenopausal women with RA [21]. In that study, those women carrying the CC genotype were more likely to develop osteoporosis than those who did not carry this genotype [21]. However, no significant association was found between the rs1805034 polymorphism in the RANK gene and both serum RANKL levels and BMD. Notoriously, unlike in our results, subjects carrying the RANK 575T allele were more likely to develop RA than those carrying other alleles.

Unfortunately, polymorphisms in the other genes studied, including sclerostin, different semaphorins (*SEMA3A*, *SEMA4B*, *SEMA4D*, *SEMA4F*, *SEMA6D*), calcitonin receptor and DKK1, did not show noteworthy alterations in any of the parameters studied.

Our study has some strengths. First, our cohort is homogeneous, with a population of Western European ancestry and very similar lifestyle habits, and quite representative of what is seen in clinical practice in a real-world setting. In addition, the follow-up was conducted in a single hospital center. Altogether, these characteristics reduce biases inherent to more heterogeneous populations. Second, the effectiveness outcomes were evaluated by two related but different measurement methods: DAS28 and HUPI (the most commonly used in our environment). The study also has several limitations. First, the study lacks data related to the presence or absence of fragility fractures in the study period. Second, data related to the quality or geometry of the bone tissue, also important in determining the quality and resistance of bone to fractures, were not collected either. Third, the relatively small sample size limits the power to detect individual effects and interactions.

## 5. Conclusions

The relationship of the immune system with bone tissue is complex and constitutes a topic of debate. In this relationship, the RANKL/RANK/OPG axis constitutes one of the key lines that favor the communication between both systems. Our study found an association between certain SNPs in genes involved in this axis and several parameters related to disease activity and BMD in a registry of patients with EA. In fact, the AA genotype in the SNPs rs3134058 and rs10505346 in the *OPG* gene is associated with higher disease activity and lower OPG serum levels at two years of follow-up. Likewise, the SNP rs4355801 located near *OPG* shows an association of the minor allele G (especially homozygote GG) with higher BMD and higher serum OPG levels. Finally, the CC genotype of rs1805034, in the *RANK* gene, is associated with low BMD values and high IL-6 levels at the baseline visit of patients included in this study. We think these data can be useful to better understand the pathogenesis of bone alteration in RA, but they must first be confirmed in larger longitudinal series.

## Figures and Tables

**Table 1:** Baseline features of patients included in the study.

Number of EA Patients	N = 268
Disease onset (years), median [IQR]	55.8 [46.2–67.8]
Females/males, n (% of females)	208/60 (77.6)
Population ethnicity, n (%)	
Western Europe (Spanish)	260 (97)
South America (Latin/Hispanic)	6 (2.2)
Eastern Europe (Slavic)	2 (0.7)
Smoking habit, n (%)	
Non-smoker	136 (50.8)
Previous smoker	66 (24.6)
Current smoker	66 (24.6)
ACPA-positive, n (%)	119 (44.9)
RF-positive, n (%)	135 (50.4)
Diagnosis, n (%)	
RA	187 (69.8)
UA	81 (30.2)
DAS28 baseline, median [IQR]	4.46 [3.35–5.55]
HUPI baseline, median [IQR]	7 [4.5–9.5]
HAQ baseline, median [IQR]	0.875 [0.5–1.6]
BMD at lumbar spine (g/cm^2^), median [IQR]	0.933 [0.836–1.041]
BMD at femoral head (g/cm^2^), median [IQR]	0.739 [0.642–0.832]
BMD at total femur (g/cm^2^), median [IQR]	0.878 [0.801–0.973]
BMD at MCP joints (g/cm^2^), median [IQR]	0.265 [0.233–0.296]
BMI (kg/m^2^), median [IQR]	26.15 [23.43–29.42]
Age menopause (years), median [IQR]	50 [46–52]

Abbreviations: n, number; IQR, interquartile range; ACPA anti-citrullinated peptide antibodies; RF, rheumatoid factor; RA, rheumatoid arthritis; UA, undifferentiated arthritis; DAS28, 28-joint Disease Activity Score; HUPI, 28-joint Disease Activity Score, Hospital Universitario de La Princesa Index; HAQ, health assessment questionnaire; BMD, bone mineral density; BMI, body mass index; MCP, metacarpophalangeal joints.

**Table 2:** Clinical parameters between groups based on DAS28 disease activity.

	Remission (DAS28 < 2.6)	Low Disease Activity (DAS28: 2.6 to 3.2)	Moderate Disease Activity (DAS28 > 3.2 to 5.1)	High Disease Activity (DAS28 > 5.1)	*p*
Number of EA patients	99	39	73	10	
Females/males, n (% of males)	68/31 (31.31%)	31/8 (20.51%)	60/13 (17.80%)	10/0 (0%)	0.047
Disease onset (years), median [IQR]	52.3 [38.8–67.9]	61 [52–70.5]	55.9 [49.8–67.5]	55.3 [51.4–62.7]	n.s.
Smoking habit, n (%)					
Non-smoker	46	22	39	4	n.s.
Former smoker	26	9	18	2	
Active smoker	27	8	16	3	
ACPA-positive, n (%)	46	18	35	3	n.s.
RF-positive, n (%)	53	19	37	3	n.s.
Diagnosis, n (%)					
RA	64	30	57	7	n.s.
UA	35	9	16	3	

Abbreviations: n, number; IQR, interquartile range; n.s., non-significant; ACPA, anti-citrullinated peptide antibodies; RF, rheumatoid factor; RA, rheumatoid arthritis; UA, undifferentiated arthritis; DAS28, 28-joint Disease Activity Score.

**Table 3:** Relationship between DAS28 and the *TNFRSF11B* genetic variant rs10505346 during the follow-up.

	β Coeff.	[95% CI]		*p*-Value
**Treatment**				
No treatment	Ref.			
MTX	−0.875	−1.036	−0.714	0.000
LEF	−0.721	−0.941	−0.501	0.000
AMs	−0.585	−0.820	−0.349	0.000
Anti-TNFα	−0.996	−1.395	−0.598	0.000
SSZ	−0.769	−1.235	−0.302	0.001
**Diagnosis**				
RA	Ref.			
UA	−0.781	−1.057	−0.541	0.000
***TNFRSF11B* (rs10505346)**				
CC	Ref.			
CA	0.103	−0.153	0.360	0.429
AA	0.597	0.027	1.167	0.040

Abbreviations: Coeff: coefficient; Ref: reference; CI: confidence interval; MTX: methotrexate; AMs: antimalarials; LEF: leflunomide; SSZ: sulfasalazine; RA: rheumatoid arthritis; UA: undifferentiated arthritis.

**Table 4:** Multivariable model of relationship between DAS28 at 2-year follow-up and the *TNFRSF11B* genetic variant rs3134058.

	β Coeff.	[95% CI]		*p*-Value
**Age at disease onset**				
<45 years	Ref.			
45–65 years	1.145	0.303	1.987	0.008
>65 years	0.808	−0.089	1.706	0.078
**Diagnosis**				
RA	Ref.			
UA	−0.418	−1.156	0.320	0.267
**ACPAs**				
Negative	Ref.			
Positive	−0.173	−0.822	0.476	0.602
***TNFRSF11B* (rs3134058)**				
GG	Ref.			
GA	0.247	−0.419	0.913	0.467
AA	0.862	0.049	1.674	0.038

Abbreviations: Coeff: coefficient; Ref: reference; RA: rheumatoid arthritis; UA: undifferentiated arthritis; ACPAs: anti-citrullinated protein antibodies.

**Table 5:** Multivariable model of relationship between DAS28 at 2-year follow-up and the rs4355801 genetic variant of *TNFRSF11B*.

	β Coeff.	[95% CI]		*p*-Value
**Gender**				
Male	Ref.			
Female	0.990	0.331	1.649	0.003
**Age at disease onset**				
<45 years	Ref.			
45–65 years	1.354	0.608	2.099	0.000
>65 years	1.125	0.332	1.918	0.005
**Diagnosis**				
RA	Ref.			
UA	−0.498	−1.165	0.170	0.144
**ACPAs**				
Negative	Ref.			
Positive	−0.376	−0.969	0.218	0.215
***TNFRSF11B* (rs4355801)**				
AA	Ref.			
AG	−0.569	−1.169	0.032	0.063
GG	−0.961	−1.729	−0.193	0.014

Abbreviations: Coeff: coefficient; Ref: reference; CI: confidence interval; RA: rheumatoid arthritis; UA: undifferentiated arthritis; ACPAs: anti-citrullinated protein antibodies.

**Table 6:** Multivariable model of relationship between hand BMD throughout the follow-up and rs1805034 genetic variant at *TNFRSF11A*.

	Β-Coeff.	[95% CI]		*p*-Value
**Age at disease onset (years) and Gender**				
<45 Male	Ref.			
<45 Female	−0.040	−0.075	−0.005	0.023
45–65 Male	−0.019	−0.057	0.018	0.314
45–65 Female	−0.065	−0.097	−0.032	0.000
>65 Male	−0.060	−0.095	−0.025	0.001
>65 Female	−0.122	−0.155	−0.089	0.000
**BMI**	0.003	0.002	0.004	0.000
***TNFRSF11A* (rs1805034)**				
AA	Ref.			
AG	−0.012	−0.023	−0.001	0.032
GG	−0.017	−0.030	−0.003	0.018

Abbreviations: Coeff: coefficient; Ref: reference; CI: confidence interval; BMI: body mass index.

## Data Availability

The datasets used and/or analyzed during the current study are available from the corresponding authors (scastas@gmail.com or amaliala@ucm.es) on reasonable request.

## Supplementary Materials

The following supporting information can be downloaded at: https://www.mdpi.com/article/10.3390/life14091109/s1. Table S1: Single-nucleotide polymorphisms (SNPs) selected in the study, chromosomal location, minor allele frequency and association with outcomes; Table S2: Multivariable model of relationship between activity at 2-year follow-up assessed by HUPI and rs3134058 in *TNFRSF11B*; Table S3: Multivariable model of relationship between activity at 2-year follow-up assessed by HUPI and Combined Genotypic Model of SNPs related to *TNFRSF11B* gene.
