# Supplementary material for: Genetic Variants in RANK and OPG Could Influence Disease Severity and Bone Remodeling in Patients with Early Arthritis
Source: Life (Basel). 2024 Sep 3;14(9):1109. doi: 10.3390/life14091109 (PMC11433004; doi:10.3390/life14091109)
Supplement: Supplementary file 1 [file life-14-01109-s001.zip › life-3135005-supplementary.pdf]

Supplementary Materials

Table S1. Single nucleotide polymorphisms (SNPs) selected in the study, chromosomal location, minor allele frequency and association with BMD and activity outcomes.

SNP	Chr	GRCh37Position	GeneSymbol	GeneLocation	MAF	Outcome BMD	Outcome Activity
**rs363609**	2	74902872	SEMA4F	INTRON	0.13		
**rs989506**	7	93147883	CALCR	INTRON	0.18		
**rs10955898**	8	119793583	LOC441377 ǀ TNFRSF11B	INTERGENIC	0.11		x
**rs4130891**	8	119921354	LOC441377 ǀ TNFRSF11B	INTERGENIC	0.18		
**rs4355801**	8	119923873	LOC441377 ǀ TNFRSF11B	INTERGENIC	0.46	x	x
**rs3134058**	8	119954108	TNFRSF11B	INTRON	0.42		x
**rs10505346**	8	119963843	TNFRSF11B	INTRON	0.22		x
**rs6469804**	8	120044829	TNFRSF11B ǀ COLEC10	INTERGENIC	0.42	x	x
**rs6993813**	8	120052238	TNFRSF11B ǀ COLEC10	INTERGENIC	0.45		x
**rs11265876**	9	92039416	LOC100133304 ǀ SEMA4D	INTERGENIC	0.36		
**rs9410482**	9	92054549	LOC100133304 ǀ SEMA4D	INTERGENIC	0.23		
**rs11265905**	9	92076595	SEMA4D ǀ LOC100128670	INTERGENIC	0.17		
**rs2498315**	9	92138399	SEMA4D ǀ GADD45G	INTERGENIC	0.32		
**rs2988066**	9	92144998	SEMA4D ǀ GADD45G	INTERGENIC	0.45		
**rs3002331**	9	92148993	SEMA4D ǀ GADD45G	INTERGENIC	0.40		
**rs3002329**	9	92151600	SEMA4D ǀ GADD45G	INTERGENIC	0.41		
**rs2480490**	9	92158225	SEMA4D ǀ GADD45G	INTERGENIC	0.40		
**rs7093925**	10	54129654	DKK1 ǀ MBL2	INTERGENIC	0.49		
**rs1194669**	10	54148289	DKK1 ǀ MBL2	INTERGENIC	0.52		
**rs17521782**	13	42898193	AKAP11 ǀ TNFSF11	INTERGENIC	0.20		
**rs9525608**	13	42898967	AKAP11 ǀ TNFSF11	INTERGENIC	0.13	x	
**rs9566959**	13	42899170	AKAP11 ǀ TNFSF11	INTERGENIC	0.30		
**rs9533067**	13	42900812	AKAP11 ǀ TNFSF11	INTERGENIC	0.14	x	
**rs9566963**	13	42902137	AKAP11 ǀ TNFSF11	INTERGENIC	0.43		
**rs9533069**	13	42904965	AKAP11 ǀ TNFSF11	INTERGENIC	0.30	x	
**rs9533070**	13	42904995	AKAP11 ǀ TNFSF11	INTERGENIC	0.19		
**rs58327984**	13	42905802	AKAP11 ǀ TNFSF11	INTERGENIC	0.44		
**rs9562396**	13	42905858	AKAP11 ǀ TNFSF11	INTERGENIC	0.29	x	
**rs17521870**	13	42907942	AKAP11 ǀ TNFSF11	INTERGENIC	0.14		
**rs9566964**	13	42908350	AKAP11 ǀ TNFSF11	INTERGENIC	0.33		

**rs151474**	13	42908680	AKAP11 ∣ TNFSF11	INTERGENIC	0.29	x	
**rs9525610**	13	42909413	AKAP11 ∣ TNFSF11	INTERGENIC	0.14	x	
**rs1555631**	13	42910063	AKAP11 ∣ TNFSF11	INTERGENIC	0.29	x	
**rs61959448**	13	42910319	AKAP11 ∣ TNFSF11	INTERGENIC	0.20		
**rs238334**	13	42910921	AKAP11 ∣ TNFSF11	INTERGENIC	0.29	x	
**rs9594729**	13	42911922	AKAP11 ∣ TNFSF11	INTERGENIC	0.15		
**rs9594731**	13	42911955	AKAP11 ∣ TNFSF11	INTERGENIC	0.14		
**rs9533078**	13	42914081	AKAP11 ∣ TNFSF11	INTERGENIC	0.20		
**rs238251**	13	42914729	AKAP11 ∣ TNFSF11	INTERGENIC	0.49		
**rs74053868**	13	42915990	AKAP11 ∣ TNFSF11	INTERGENIC	0.11		x
**rs238253**	13	42916029	AKAP11 ∣ TNFSF11	INTERGENIC	0.14	x	
**rs9533083**	13	42917069	AKAP11 ∣ TNFSF11	INTERGENIC	0.38		
**rs17063206**	13	42917459	AKAP11 ∣ TNFSF11	INTERGENIC	0.14		x
**rs77900571**	13	42917860	AKAP11 ∣ TNFSF11	INTERGENIC	0.32		
**rs9525613**	13	42918648	AKAP11 ∣ TNFSF11	INTERGENIC	0.29		
**rs77565961**	13	42919194	AKAP11 ∣ TNFSF11	INTERGENIC	0.13		x
**rs9525614**	13	42920520	AKAP11 ∣ TNFSF11	INTERGENIC	0.19	x	
**rs4614629**	13	42920990	AKAP11 ∣ TNFSF11	INTERGENIC	0.19	x	
**rs17063218**	13	42922259	AKAP11 ∣ TNFSF11	INTERGENIC	0.23		
**rs17522044**	13	42923203	AKAP11 ∣ TNFSF11	INTERGENIC	0.19	x	
**rs238255**	13	42923361	AKAP11 ∣ TNFSF11	INTERGENIC	0.42		
**rs7322303**	13	42924647	AKAP11 ∣ TNFSF11	INTERGENIC	0.19	x	
**rs238257**	13	42924883	AKAP11 ∣ TNFSF11	INTERGENIC	0.38	x	
**rs9594733**	13	42925732	AKAP11 ∣ TNFSF11	INTERGENIC	0.14		
**rs7999972**	13	42930322	AKAP11 ∣ TNFSF11	INTERGENIC	0.28	x	
**rs7995775**	13	42933413	AKAP11 ∣ TNFSF11	INTERGENIC	0.28	x	
**rs238273**	13	42934017	AKAP11 ∣ TNFSF11	INTERGENIC	0.33		
**rs238274**	13	42934627	AKAP11 ∣ TNFSF11	INTERGENIC	0.32		
**rs238275**	13	42934811	AKAP11 ∣ TNFSF11	INTERGENIC	0.32		
**rs11617713**	13	42937043	AKAP11 ∣ TNFSF11	INTERGENIC	0.28	x	
**rs9525616**	13	42938839	AKAP11 ∣ TNFSF11	INTERGENIC	0.46		
**rs9594735**	13	42939932	AKAP11 ∣ TNFSF11	INTERGENIC	0.45		
**rs9594736**	13	42940207	AKAP11 ∣ TNFSF11	INTERGENIC	0.43		
**rs386283**	13	42942263	AKAP11 ∣ TNFSF11	INTERGENIC	0.12		
**rs7992970**	13	42945463	AKAP11 ∣ TNFSF11	INTERGENIC	0.27		
**rs71429414**	13	42945821	AKAP11 ∣ TNFSF11	INTERGENIC	0.23		
**rs9525617**	13	42947427	AKAP11 ∣ TNFSF11	INTERGENIC	0.19		
**rs34211487**	13	42947585	AKAP11 ∣ TNFSF11	INTERGENIC	0.22		
**rs1886214**	13	42948531	AKAP11 ∣ TNFSF11	INTERGENIC	0.23		
**rs2209241**	13	42948660	AKAP11 ∣ TNFSF11	INTERGENIC	0.16		x
**rs1886215**	13	42949359	AKAP11 ∣ TNFSF11	INTERGENIC	0.46		
**rs17063269**	13	42950035	AKAP11 ∣ TNFSF11	INTERGENIC	0.18		
**rs9533087**	13	42950320	AKAP11 ∣ TNFSF11	INTERGENIC	0.19		
**rs12871509**	13	42952075	AKAP11 ∣ TNFSF11	INTERGENIC	0.26		
**rs9594738**	13	42952145	AKAP11 ∣ TNFSF11	INTERGENIC	0.43	x	

**rs17457484**	13	42952499	AKAP11 ∣ TNFSF11	INTERGENIC	0.27		
**rs56008941**	13	42952611	AKAP11 ∣ TNFSF11	INTERGENIC	0.27		
**rs1475248**	13	42953003	AKAP11 ∣ TNFSF11	INTERGENIC	0.26		
**rs989354**	13	42953336	AKAP11 ∣ TNFSF11	INTERGENIC	0.26		
**rs1475249**	13	42954505	AKAP11 ∣ TNFSF11	INTERGENIC	0.31		
**rs12870516**	13	42955965	AKAP11 ∣ TNFSF11	INTERGENIC	0.27		
**rs11840862**	13	42956463	AKAP11 ∣ TNFSF11	INTERGENIC	0.31	x	
**rs35246711**	13	42956657	AKAP11 ∣ TNFSF11	INTERGENIC	0.32		
**rs1408222**	13	42956875	AKAP11 ∣ TNFSF11	INTERGENIC	0.26		
**rs35406824**	13	42957324	AKAP11 ∣ TNFSF11	INTERGENIC	0.27		
**rs8001611**	13	42965694	AKAP11 ∣ TNFSF11	INTERGENIC	0.43	x	
**rs9533094**	13	42965837	AKAP11 ∣ TNFSF11	INTERGENIC	0.42	x	
**rs77855024**	13	42971615	AKAP11 ∣ TNFSF11	INTERGENIC	0.12		
**rs1324004**	13	42974209	AKAP11 ∣ TNFSF11	INTERGENIC	0.26		
**rs1324003**	13	42976126	AKAP11 ∣ TNFSF11	INTERGENIC	0.31		
**rs12853439**	13	42978035	AKAP11 ∣ TNFSF11	INTERGENIC	0.26		
**rs12865514**	13	42979444	AKAP11 ∣ TNFSF11	INTERGENIC	0.28		
**rs2324878**	13	42980878	AKAP11 ∣ TNFSF11	INTERGENIC	0.28		
**rs9566973**	13	42986541	AKAP11 ∣ TNFSF11	INTERGENIC	0.25		
**rs4942114**	13	42986731	AKAP11 ∣ TNFSF11	INTERGENIC	0.26		
**rs9533099**	13	42987357	AKAP11 ∣ TNFSF11	INTERGENIC	0.26		
**rs9594745**	13	42987938	AKAP11 ∣ TNFSF11	INTERGENIC	0.26		
**rs7327510**	13	42988924	AKAP11 ∣ TNFSF11	INTERGENIC	0.46		
**rs17638696**	13	42989440	AKAP11 ∣ TNFSF11	INTERGENIC	0.18		
**rs9562401**	13	42990400	AKAP11 ∣ TNFSF11	INTERGENIC	0.25		
**rs2324877**	13	42991060	AKAP11 ∣ TNFSF11	INTERGENIC	0.25		
**rs9590697**	13	42994481	AKAP11 ∣ TNFSF11	INTERGENIC	0.27		
**rs9533100**	13	42996548	AKAP11 ∣ TNFSF11	INTERGENIC	0.45		
**rs9525620**	13	42996632	AKAP11 ∣ TNFSF11	INTERGENIC	0.25		
**rs9533101**	13	42996797	AKAP11 ∣ TNFSF11	INTERGENIC	0.25		
**rs6561043**	13	42997574	AKAP11 ∣ TNFSF11	INTERGENIC	0.28		
**rs4942120**	13	43005533	AKAP11 ∣ TNFSF11	INTERGENIC	0.30		x
**rs1924416**	13	43008994	AKAP11 ∣ TNFSF11	INTERGENIC	0.29		
**rs9533103**	13	43010483	AKAP11 ∣ TNFSF11	INTERGENIC	0.13		
**rs4942121**	13	43012590	AKAP11 ∣ TNFSF11	INTERGENIC	0.27		
**rs9533104**	13	43017895	AKAP11 ∣ TNFSF11	INTERGENIC	0.43		
**rs9525625**	13	43018030	AKAP11 ∣ TNFSF11	INTERGENIC	0.45		
**rs4942122**	13	43018898	AKAP11 ∣ TNFSF11	INTERGENIC	0.25		
**rs2147160**	13	43021639	AKAP11 ∣ TNFSF11	INTERGENIC	0.31		x
**rs9315919**	13	43029068	AKAP11 ∣ TNFSF11	INTERGENIC	0.28		
**rs41482750**	13	43030937	AKAP11 ∣ TNFSF11	INTERGENIC	0.33	x	
**rs12430303**	13	43032027	AKAP11 ∣ TNFSF11	INTERGENIC	0.43		
**rs9594759**	13	43032593	AKAP11 ∣ TNFSF11	INTERGENIC	0.48		
**rs1974021**	13	43033102	AKAP11 ∣ TNFSF11	INTERGENIC	0.19		
**rs9533109**	13	43033187	AKAP11 ∣ TNFSF11	INTERGENIC	0.22		

**rs17457881**	13	43034284	AKAP11 | TNFSF11	INTERGENIC	0.26		
**rs1853573**	13	43034968	AKAP11 | TNFSF11	INTERGENIC	0.48		
**rs9594766**	13	43040043	AKAP11 | TNFSF11	INTERGENIC	0.45		
**rs1886211**	13	43040438	AKAP11 | TNFSF11	INTERGENIC	0.25		
**rs7990020**	13	43044946	AKAP11 | TNFSF11	INTERGENIC	0.46		
**rs2093816**	13	43046036	AKAP11 | TNFSF11	INTERGENIC	0.47		
**rs927623**	13	43046054	AKAP11 | TNFSF11	INTERGENIC	0.25		
**rs7322806**	13	43046397	AKAP11 | TNFSF11	INTERGENIC	0.25		
**rs9533117**	13	43046812	AKAP11 | TNFSF11	INTERGENIC	0.30		
**rs9594768**	13	43049482	AKAP11 | TNFSF11	INTERGENIC	0.49		
**rs2062305**	13	43052880	AKAP11 | TNFSF11	INTERGENIC	0.45		
**rs35486210**	13	43054684	AKAP11 | TNFSF11	INTERGENIC	0.27		
**rs34132030**	13	43056036	AKAP11 | TNFSF11	INTERGENIC	0.30		
**rs1351832**	13	43057549	AKAP11 | TNFSF11	INTERGENIC	0.31		x
**rs9594770**	13	43058031	AKAP11 | TNFSF11	INTERGENIC	0.32		
**rs17536071**	13	43060407	AKAP11 | TNFSF11	INTERGENIC	0.27		
**rs2046387**	13	43063646	AKAP11 | TNFSF11	INTERGENIC	0.22		
**rs12868231**	13	43064910	AKAP11 | TNFSF11	INTERGENIC	0.29		
**rs9533128**	13	43065404	AKAP11 | TNFSF11	INTERGENIC	0.15		
**rs9533129**	13	43066117	AKAP11 | TNFSF11	INTERGENIC	0.32		
**rs4942132**	13	43069998	AKAP11 | TNFSF11	INTERGENIC	0.23		
**rs4942133**	13	43070050	AKAP11 | TNFSF11	INTERGENIC	0.22		
**rs35860234**	13	43070206	AKAP11 | TNFSF11	INTERGENIC	0.22		
**rs9315922**	13	43070780	AKAP11 | TNFSF11	INTERGENIC	0.22		
**rs9315923**	13	43070894	AKAP11 | TNFSF11	INTERGENIC	0.44		
**rs66880203**	13	43072531	AKAP11 | TNFSF11	INTERGENIC	0.16		
**rs10507510**	13	43073157	AKAP11 | TNFSF11	INTERGENIC	0.21		
**rs12869389**	13	43073459	AKAP11 | TNFSF11	INTERGENIC	0.33		
**rs17536113**	13	43083876	AKAP11 | TNFSF11	INTERGENIC	0.34		
**rs9533133**	13	43083934	AKAP11 | TNFSF11	INTERGENIC	0.41		
**rs346598**	13	43084125	AKAP11 | TNFSF11	INTERGENIC	0.20		
**rs346599**	13	43084305	AKAP11 | TNFSF11	INTERGENIC	0.23		
**rs56967493**	13	43084877	AKAP11 | TNFSF11	INTERGENIC	0.44		
**rs35111182**	13	43085198	AKAP11 | TNFSF11	INTERGENIC	0.36		
**rs7336228**	13	43085216	AKAP11 | TNFSF11	INTERGENIC	0.22		
**rs4941430**	13	43086351	AKAP11 | TNFSF11	INTERGENIC	0.23		
**rs4941431**	13	43086478	AKAP11 | TNFSF11	INTERGENIC	0.19		
**rs376999**	13	43086646	AKAP11 | TNFSF11	INTERGENIC	0.22		
**rs3862738**	13	43086907	AKAP11 | TNFSF11	INTERGENIC	0.32	x	
**rs9562406**	13	43097559	AKAP11 | TNFSF11	INTERGENIC	0.19	x	
**rs4942139**	13	43098328	AKAP11 | TNFSF11	INTERGENIC	0.21		
**rs35230292**	13	43098967	AKAP11 | TNFSF11	INTERGENIC	0.22		
**rs9533139**	13	43099648	AKAP11 | TNFSF11	INTERGENIC	0.41		
**rs568215**	15	48063076	SEMA6D	UTR	0.40		

**rs1513670**	17	41807331	LOC100128016 | SOST	INTERGENIC	0.40	x	
**rs1805034**	18	60027241	TNFRSF11A	CODING	0.44	x	
**rs4426449**	18	60042817	TNFRSF11A	INTRON	0.34		
**rs2957145**	18	60076799	TNFRSF11A | ZCCHC2	INTERGENIC	0.37		
**rs599323**	18	60165701	TNFRSF11A | ZCCHC2	INTERGENIC	0.21		x

Abbreviations: SNPs: single nucleotide polymorphisms; Chr: chromosome; MAF: minor allele frequencies; BMD: bone mineral density; x: association with outcome with p<0.05.

Table S2. Multivariable model of relationship between activity at 2-year follow-up assessed by HUPI and rs3134058 in *TNFRSF11B.*

	**B-Coeff.**	**Std. Err.**	**P>|z|**	**[95% Conf. Interval]**	
**Age of disease onset, years**					
< 45	Ref.				
45 - 65	1.199	0.755	0.001	0.463	1.935
> 65	1.033	0.398	0.009	0.253	1.813
**Diagnosis**					
RA	Ref.				
UA	-0.446	0.330	0.177	-1.093	0.201
**ACPA**					
Negative	Ref.				
Positive	-0.167	0.291	0.567	-0.737	0.404
***TNFRSF11B* (rs3134058)**					
GG	Ref.				
GA	0.497	0.274	0.070	-0.040	1.034
AA	1.551	0.658	0.018	0.260	2.841

Abbreviations: HUPI: hospital universitario de la Princesa index; Coeff: coefficient; Ref: reference; CI: confidence interval; RA: rheumatoid arthritis; UA: undifferentiated arthritis; ACPA: anti-citrullinated protein antibodies.

Table S3. Multivariable model of relationship between activity at 2-year follow-up assessed by HUPI and Combined Genotypic Model of SNPs related to *TNFRSF11B* gene.

	B-Coeff.	Std. Err.	P>|z|	[95% Conf. Interval]	
**Age of disease onset, years**					
< 45	Ref.				
45 - 65	0.181	0.573	0.752	-0.942	1.305
> 65	-0.021	0.595	0.972	-1.187	1.145
**Diagnosis**					
RA	Ref.				
UA	-1.635	0.602	0.007	-2.815	-0.456
**ACPA**					
Negative	Ref.				
Positive	-0.569	0.443	0.199	-1.437	0.299
**CGM**					
0	Ref.				
1	0.570	0.446	0.201	-0.304	1.444
2	1.901	0.742	0.010	0.447	3.355

Abbreviations: HUPI: hospital universitario de la Princesa index; SNPs: single nucleotide polymorphisms; Coeff: coefficient; Ref: reference; CI: confidence interval; RA: rheumatoid arthritis; UA: undifferentiated arthritis; ACPA: anti-citrullinated protein antibodies, CGM: Combined Genotypic Model (CGM=0 for patients who are carriers of CC-rs10505346, GG-rs3134058 and GG-rs4355801; CGM=2 for patients who are carriers of AA-rs10505346, AA-rs3134058 and AA-rs4355801 and CGM=1 for patients heterozygotes of each SNP).
